# Cellular Functions of High-Temperature Requirement Factor A4 in Placenta

**DOI:** 10.3390/cells12111459

**Published:** 2023-05-24

**Authors:** Chang-Zhu Pei, Bum-Chae Choi, Jun-Hyeok Park, Hyo Young Park, Jinyoung Paek, Kyung-Ju Lee, Bo-Seong Yun, Young Ju Kim, Kwang-Hyun Baek

**Affiliations:** 1 Department of Biomedical Science, Cell and Gene Therapy Research Institute, CHA University, Seongnam 13488, Republic of Korea; czpei1983@163.com (C.-Z.P.); nymwns1@naver.com (J.-H.P.); 2 Department of Obstetrics and Gynecology, CL Women’s Hospital, Gwangju 61917, Republic of Korea; cbcmd60@gmail.com (B.-C.C.); hytwyb@gmail.com (H.Y.P.); 3 Department of Laboratory Medicine, Gangnam CHA Hospital, College of Medicine, Seoul 06135, Republic of Korea; jyoung@cha.ac.kr; 4 Department of Obstetrics and Gynecology, Korea University Anam Hospital, Korea University College of Medicine, Seoul 02841, Republic of Korea; drlkj52551@korea.ac.kr; 5 Department of Obstetrics and Gynecology, Ilsan CHA Hospital, College of Medicine, Seoul 06135, Republic of Korea; bosungyun@chamc.co.kr; 6 Department of Obstetrics and Gynecology, Ewha Woman’s University College of Medicine, Seoul 07985, Republic of Korea; kkyj@ewha.ac.kr

**Keywords:** biomarker, cellular functions, CRISPR/Cas9, HtrA4, recurrent abortion

## Abstract

The expression of *High-temperature requirement factor A4 (HtrA4)* mRNA is significantly lower in the chorionic villi of patients with recurrent pregnancy loss (RPL) than in the control group. We conducted an investigation into the cellular functions of HtrA4 using the CRISPR/Cas9 system and shRNA-*HtrA4* to create knockout BeWo cells and *HtrA4* knockdown JEG3 cells. Our results indicated that the knockout BeWo cells exhibited reduced capacity for invasion and fusion, but increased levels of proliferation and migration, with a significantly shortened cell cycle compared to wild-type cells. Wild-type BeWo cells highly expressed cell invasion- and fusion-related factors, while knockout BeWo cells highly expressed migration-, proliferation-, and cell cycle-related factors. The shRNA-*HtrA4* JEG3 cells showed a decreased capacity for invasion, but an increased capacity for migration, accompanied by a decrease in the expression of cell invasion-related factors and an increase in migration-related factors. Moreover, our ELISA results revealed that the serum HtrA4 level was lower in patients with RPL than in the controls. These findings suggest that HtrA4 depletion may be associated with placental dysfunction.

## 1. Introduction

Members of the high-temperature requirement A (HtrA) family of serine proteases include HtrA1, HtrA2, HtrA3, and HtrA4 [1]. HtrA4 is a serine protease with proteolytic activity [2]. HtrA4 contains an enzymatically active serine protease domain and a protein-protein interaction PDZ domain [3]. HtrA4 mainly exists in the trimeric form and hydrolyzes the substrate [3]. The structure of the HtrA4 domain is highly comparable to that of HtrA1 and HtrA3, and several publications have reported that the HtrA4 as a tumor suppressor is involved in oncogenesis [4]. Kummari et al. reported that HtrA4 influences cell apoptosis via the degradation of XIAP [3]. In addition, Wenta et al. reported that HtrA4 might cleave XIAP, caspase 9, and caspase 7 to influence apoptosis [2]. While the proteolytic activity of HtrA4 is relatively low, the decomposition of XIAP by HtrA4 alone may not be sufficient to induce apoptosis [2]. Therefore, it is probable that the decomposition of XIAP by HtrA4 requires additional pro-apoptotic processes to occur concurrently in order to trigger apoptosis [2]. Wenta et al. reported that HtrA4 facilitates apoptosis in etoposide-treated A549 cells and enhances blockage of the cell cycle at the G2/M phase to decrease cell growth [4]. The HtrA4 interacts with several substrates and performs various functions in different cells. The upregulation of HtrA4 in preeclamptic circulation cleaves the main VEGFA receptor KDR and a key junctional protein VE-cadherin, which may disrupt endothelial cell function and the endothelial barrier [5,6]. In addition, the upregulation of HtrA4 leads to inflammatory responses in human umbilical vein endothelial cells (HUVECs) by increasing pro-inflammatory related genes *IL-6*, *PTGS2* (*COX2*), and *IL-1β* [7]. HtrA4, as a serine protease, is involved in trophoblast molecular functions [8]. One of the downstream target genes for glial cells missing transcription factor 1 (GCM1) is *HtrA4*, which is involved in placental development by regulating the expression of HtrA4 [9]. Wang et al. reported that a receptor for activated C-kinase 1 upregulates the stability of GCM1 to influence placental cell invasion and migration [10]. Chiu et al. reported that GATA3 interacts with GCM1 to inhibit its activity and influence placental cell invasion [11].

HtrA4 is a protein that is secreted and its level in the serum varies during different stages of pregnancy [12]. It has been shown that the serum level of HtrA4 gradually increases between 11–13 weeks and 24–25 weeks of gestation, and then remains stable during the remainder of gestation [12]. Several studies have demonstrated that HtrA4 is highly expressed in the sera of patients with preeclampsia, and can affect the invasion and migration of trophoblasts, leading to this condition [6,9,13]. In addition, HtrA4 has been upregulated in breast carcinoma [14], while it is downregulated in glioblastoma [15], pancreatic cancer [16], and pediatric disease compared to normal samples [17]. Moreover, Varambally et al. reported that the expression of HtrA4 is lower in hormone-refractory metastatic prostate cancer than in primary prostate carcinoma [18].

In our previous study, we performed a subtractive hybridization analysis to compare the differential gene expressions in chorionic villi from patients with recurrent pregnancy loss (RPL) and control groups [19]. We identified eight genes related to immunosuppression, embryo attachment, and angiogenesis, including *HtrA4*, which was an unknown gene at that time [19]. The mRNA level of *HtrA4* in RPL patients was significantly lower than values obtained in normal women during the 6th and 8th weeks of gestation [19]. The trophoblast is a native placental tissue that plays a critical role in placental development, including appropriate fusion, proliferation, differentiation, migration, angiogenesis, invasion, and adhesion [20]. Various growth factors, cytokines, enzymes, hormones, and proteins regulate these phases of placenta development [21]. Aberrant expression of these molecules can result in placental dysfunction and the associated complications of pregnancy, such as miscarriage, preeclampsia, fetal growth restriction, and molar pregnancies [21,22,23].

Although the roles of HtrA4 have been extensively studied, the effect of the direct knockout or knockdown of the *HtrA4* gene on trophoblast cells is not yet clear. The aim of this study is to investigate the effects of HtrA4 on the cell fusion, invasion, migration, proliferation, and cell cycle in BeWo and JEG3 cells.

## 2. Materials and Methods

### 2.1. Subjects and Samples

This study involved RPL patients and normal controls who visited the Fertility Center of the CHA General Hospital in the Republic of Korea. All participants provided informed written consent, and the study, which involved human blood samples, was authorized by the Ethics Committee of CHA University located in Seoul, the Republic of Korea (Reference Number: 08-16, 1044308-201805-BR-023-02). RPL patients were selected based on the number of prior losses (*n* = 2, 3, or ≥4) and underwent several evaluations, including karyotyping for both partners, hysterosalpingography, hysteroscopy, blood tests for RPL, TSH, fasting glucose, mid-luteal P4, lupus anticoagulant activity, dilute Russell viper venom test, PT/APTT, IgG, IgM-anticardiolipin, antiphosphatidylserine antibodies, cervical cultures for Chlamydia, mycoplasma, and ureaplasma, as well as assessments for the frequency of abnormal results in functional protein C activity, functional protein S activity, fasting homocysteine, antithrombin III, Factor V Leiden, antithyroglobulin antibody, anti-ß-GPI IgG, and *methylenetetrahydrofolatereductase* (*MTHFR*) gene polymorphisms. Only RPL patients who did not show abnormal results from the above evaluations were included in the study, while the normal controls were women with no history of miscarriage and obstetric complications (Table 1). Blood samples were collected from both normal controls and RPL patients with regular menstrual cycles on the 5th to 9th days after ovulation in the menstrual cycle.

### 2.2. Cell Culture

The BeWo cell line (KCLB NO 10098) was purchased from Republic of Korea Cell Line Bank, while the JEG3 (ATCC Number: HTB-36), HTR-8/SVneo (ATCC Number: CRL-3271), and HEK293 (ATCC Number: CRL-3216) cell lines were obtained from the American Type Culture Collection (ATCC, Manassas, VA, USA). Wild-type BeWo (BeWo WT), *HtrA4* gene knockout BeWo (BeWo KO), *HtrA4*-rescued BeWo KO (BeWo KO-*HtrA4* rescue), as well as JEG3 and HTR-8/SVneo cells, were cultured in Dulbecco’s Modified Eagle’s Medium (DMEM, Gibco-BRL, Rockville, MD, USA), supplemented with 10% fetal bovine serum (FBS, Gibco, Grand Island, NY, USA) and 1% penicillin-streptomycin (FBS, Gibco, Grand Island, NY, USA). The cells were incubated in a humidified incubator with 5% CO_2_ at 37 °C.

### 2.3. Construction of Expression Vectors

The pcDNA3.1-*HtrA4*-Flag plasmid was obtained from Prof. Hungwen Chen (National Taiwan University, Taiwan). For RNA interference, pSilencer 3.1-H1 neo short-hairpin RNA (shRNA) containing the *HtrA4* (5′-AAG CTA CAT ACC CAG CCC TCC-3′) sequences was used [9]. To transfect the pcDNA3.1-*HtrA4*-Flag plasmid, 3 μg of the plasmid was added to 1 × 10^6^ BeWo KO cells using Lipofectamine 2000 (Invitrogen, Carlsbad, CA, USA), and the cells were incubated for 48 h. The expression of HtrA4 was analyzed using whole cell lysates and western blot analysis. For the transfection of shRNA-*HtrA4*, 3 μg of the plasmid was added to 1 × 10^6^ JEG3 cells using Lipofectamine 2000 (Invitrogen, Carlsbad, CA, USA), and the transfection efficiency was examined using western blotting.

### 2.4. Generating HtrA4 Knockout in BeWo Cell Line by the CRISPR/Cas9 System

The pSpCas9 (BB)-2A-GFP (pX458) plasmid was purchased from Addgene (Watertown, MA, USA), and the *HtrA4*-sgRNA was designed from CRISPR RGEN Tools (http://www.rgenome.net accessed on 3 August 2018) (Table S1) and cloned into the pX458 vector. The BeWo WT cells were transfected with *HtrA4*-sgRNA-pX458 using Lonza 4D-Nucleofector (Lonza, Cologne, Germany) according to the manufacturer’s instructions. The details of *HtrA4*-sgRNA-pX458 were shown in Figure S1A–C. The *HtrA4*-sgRNA-pX458 plasmid expresses sgRNA targeting the *HtrA4* gene, Cas9 protein, and GFP (which expresses green fluorescence). The transfected polyclonal cells were observed for transfection efficiency under a fluorescence microscope. To select monoclonal cell lines, the polyclonal cells were seeded at a density of one cell per well in a 96-well plate, and the wells with one fluorescent cell were marked. In this study, we marked three wells, and one cell was cultured to a monoclonal cell line, starting with a 96-well plate and transferring to 48-well, 24-well, 12-well, 6-well plates, and finally to a 6 cm culture dish. Genomic DNAs were extracted from BeWo WT and BeWo KO cells using the Accuprep Genomic DNA Extraction Kit (Bioneer Corporation, Daejeon, Republic of Korea) as per the manufacturer’s instructions. The *HtrA4*-T7E1 primers (Table S2) were designed for polymerase chain reaction (PCR) using genomic DNA to confirm whether the target gene was knocked out. The T7 endonuclease 1 (T7E1) assay was performed using the PCR products to select the knockout cell lines. To confirm whether the *HtrA4* gene was knocked out, TA cloning (RBC Bioscience, Taipei, Taiwan) was performed using cells identified by the T7E1 assay.

### 2.5. Reverse Transcriptase-Polymerase Chain Reaction (RT-PCR)

Total RNA was isolated from 1 × 10^6^ BeWo WT and BeWo KO cells using the TRIzol^TM^ (Invitrogen, Carlsbad, CA, USA). The cDNA was synthesized using the cDNA synthesis kit (COSMOGENETECH Inc., Seoul, Republic of Korea) and 2 μg of total RNA samples. The PCR conditions were as follows: initial melting at 95 °C, 5 min, followed by 35 cycles of amplification at 95 °C for 20 s, 55 °C for 20 s, and 72 °C for 30 s, and a final extension at 72 °C for 2 min. The primer information for *HtrA4* is provided in Table S2.

### 2.6. Cell-Cell Fusion Analysis

Glass coverslips were placed in a 24-well plate and 2 × 10^4^ cells/500 μL of BeWo WT, BeWo KO, and BeWo KO-*HtrA4* rescue cells were seeded. The cells were exposed to 50 μM forskolin or equal amounts of DMSO for 48 h. After treatment, the cells were fixed using 4% paraformaldehyde for 10 min at room temperature (RT) and washed twice with phosphate-buffered saline (PBS) for 2 min each time. The cells were then permeabilized with 0.2% triton X-100 for 1 min at RT, blocked with PBS containing 1% BSA for 1 h at RT, and incubated with E-cadherin and HCG antibodies (Table S3) at 4 °C overnight. After washing with PBS, the cells were incubated with Alexa-fluor-488-conjugated donkey anti-goat antibody (Invitrogen, Carlsbad, CA, USA) and Alexa-fluor-568-conjugated goat anti-rabbit antibody (Invitrogen, Carlsbad, CA, USA) at RT for 1 h. The cell-cell fusion was examined under a confocal microscopy (Zeiss LSM880, Carl Zeiss Microscopy GmbH, Munich, Germany).

### 2.7. Invasion Assay In Vitro

A total of 200 μL of BeWo WT, BeWo KO, BeWo KO-*HtrA4* rescue, JEG3, and shRNA-*HtrA4* JEG3 cells were seeded at a density of 1 × 10^4^ cell/mL into the 24-well upper chamber (8.0 μm pore membranes; BD Biosciences, Franklin Lakes, NJ, USA) using the fetal bovine serum-free medium. The lower chamber was filled with 750 μL of medium with 10% FBS. After incubation for 48 h at 37 °C, the non-invasive cells were removed from the upper chamber using cotton swabs. The cells in the lower membrane surface were stained with crystal violet for 10 s to identify invasive BeWo and JEG3 cells. The invasive chamber was washed twice with PBS, and the number of invaded BeWo and JEG3 cells were counted under the microscope (Olympus, Tokyo, Japan).

### 2.8. Scratch Wound Assay In Vitro

To account for the effect of cell proliferation activity on cell migration, BeWo WT, BeWo KO, and BeWo KO-*HtrA4* rescue cells were treated with 2 mM thymidine for 16 h and cultured until 70~80% confluence. The BeWo and JEG3 cells were scraped in a straight line using a 200 μL or 1000 μL micropipette tip, and washed with PBS to remove stripped cells. Digital photographs were taken at 0, 24, and 48 h and analyzed using the Image J software (National Institutes of Health, Bethesda, MD, USA, version 1.4.3.67). The photographs were captured using a microscope (Olympus, Tokyo, Japan).

### 2.9. Cell Proliferation Assays

BeWo WT, BeWo KO, and BeWo KO-*HtrA4* rescue cells were seeded into 96-well plates at a density of 1 × 10^3^ cells per well and incubated for 0, 1, 2, 3, 4, and 5 days for the cell counting kit-8 (CCK-8) assay (DOJINDO Inc., Rockville, MD, USA). JEG3 and shRNA-*HtrA4* JEG3 cells were seeded into 96-well plates at a density of 5 × 10^2^ cells per well and incubated for 0, 2, 4, 6, and 8 days for the CCK-8 assay. For the colony-forming unit (CFU) assay, 1 × 10^3^ JEG3 or 1 × 10^2^ BeWo cells were seeded into 100 mm dishes and incubated with 5% CO_2_ at 37 °C for 10 days. The CCK-8 and CFU assays were performed as previously described [24].

### 2.10. Cell Cycle Analysis and Flow Cytometry

BeWo WT and BeWo KO cells were plated at 40–50% confluence in 100 mm dishes (1.5 × 10^6^ cell per dish). BeWo WT cells were incubated for 0, 3, 6, 9, 12, 15, 18, 21, 24, 27, 30, 33, 36, 39 42, 45, and 48 h, while BeWo KO cells were incubated for 0, 3, 6, 9, 12, 15, 18, 21, 24, and 27 h. The collected cells were washed with pre-cold PBS, fixed using cold 70% ethanol for 30 min, and washed twice with pre-cold PBS. Cells were then stained with FxCycle^TM^ PI/RNase staining solution RT in the dark for at least 30 min, followed by analysis on a Cyto FLEX Flow Cytometer (Beckman Coulter, Brea, CA, USA). The data were analyzed using CytExpert software (Beckman Coulter, Inc., Brea, CA, USA, version 2.3.0.84).

### 2.11. Western Blot Analysis

To obtain whole cell lysates, cells were washed in PBS and lysed on ice in 50 mM of Tris, 150 mM of NaCl, pH 7.4, 1 mM of EDTA, 1% Triton X-10, 10% glycerol, 0.1 mM of phosphatase inhibitors (Na3VO4), protease inhibitor cocktail and 1 mM of phenylmethylsulfonyl fluoride. The lysates were centrifuged at 4 °C at 16,200× *g* for 20 min, and the protein concentration was estimated using the Bradford reagent (BioRad, Hercules, CA, USA). Samples with equal amounts of protein were loaded onto a 12% SDS-PAGE gel and transferred onto a polyvinylidene fluoride (PVDF) microporous membrane (Millipore, Billerica, MA, USA). The membranes were blocked for 1 h with 5% skim milk (cat. no. 232100; Becton, Dickinson and Company, Franklin Lakes, NJ, USA) or bovine serum albumin (BSA) (cat. no. BSAS 0.1; Bovogen Biologicals, Keilor East, VIC, Australia) in TTBS (20 mM of Tris–HCl, pH 7.5, 0.05% Tween 20, 150 mM of NaCl) and incubated with the primary antibody (Table S3) at 4 °C overnight. The membranes were then incubated with the secondary antibodies goat anti-mouse IgG (H + L) (LGC SeraCare, Milford, MA, USA) or goat anti-rabbit IgG (H + L) (Santa Cruz Biotechnology, Santa Cruz, CA, USA) at RT for 1 h. The membrane was detected using an ECL reagent solution (Young-In Frontier, Seoul, Republic of Korea). The primary antibodies were diluted with 2% skim milk or BSA. The band density of western blots was quantified using the Image J software (National Institutes of Health, Bethesda, MD, USA, version 1.4.3.67), and the gray scale values of the target protein were normalized to that of β-actin.

### 2.12. ELISA for HtrA4

The serum HtrA4 level was measured using an HtrA4 ELISA kit (Uscn Life Science Inc., Wuhan, China) following the manufacturer’s instructions. Duplicate measurements were taken for all serum samples. Both inter- and intra-assay variations were less than 10%.

### 2.13. Statistical Analysis

Data were presented as means ± standard deviation (SD). Statistical analyses were conducted using GraphPad Prism 5.0 software (GraphPad Software, La Jolla, CA, USA, version 5.01) using either Student’s *t*-test or one-way ANOVA, followed by Tukey’s post hoc test. Cell counting and densitometric analysis were performed using Image J software (National Institutes of Health, Bethesda, MD, USA, version 1.4.3.67). All data were obtained from a minimum of three independent experiments. *p* < 0.05 (*), *p* < 0.01 (**), or *p* < 0.001 (***) were considered statistically significant.

## 3. Results

### 3.1. Generation of HtrA4 Knockout in BeWo Cell Line Using CRISPR/Cas9 System

**Figure 1:**
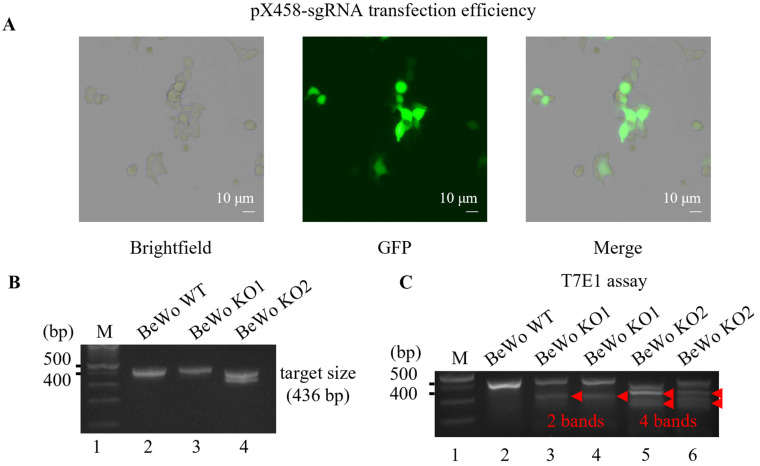

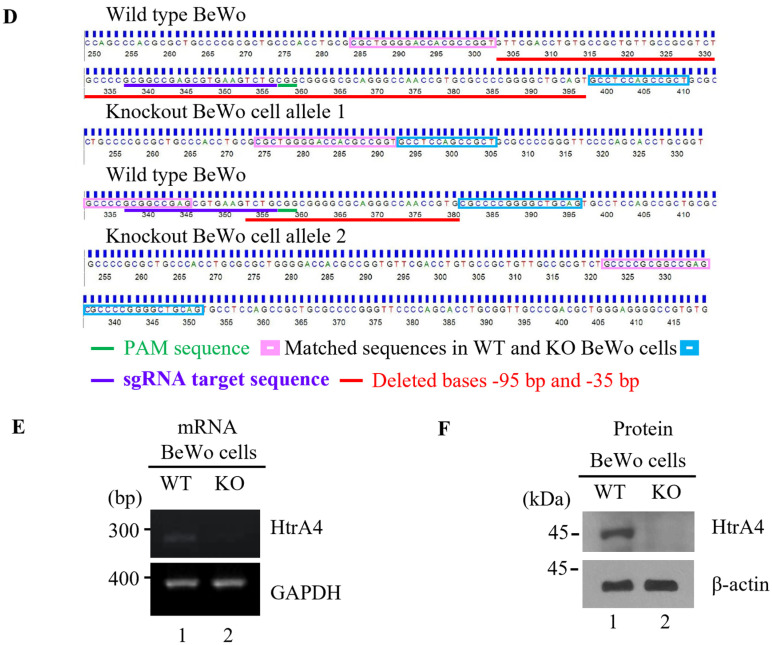
Generation of *HtrA4* knockout in BeWo cells. (**A**) BeWo WT cells were transfected with *HtrA4*-sgRNA-pX458 plasmid through electroporation. After 36 h, the transfection efficiency was determined using fluorescent microscopy. (**B**) *HtrA4* T7E1 primers were utilized to amplify PCR products containing the sgRNA target in both BeWo WT and BeWo KO cells. (**C**) The T7E1 assay was performed on the PCR products to detect the cleavage of the DNA. (**D**) Direct sequencing of PCR products was performed on both alleles 1 and 2. The sgRNA target site is underlined in purple, PAM sequences are underlined in green, deletion sequences are underlined in red, co-present sequences near 5′ primer are framed in pink, and those near 3′ primer are framed in blue. This fragment contains all the components required for gRNA expression. RT-PCR and western blot analysis, respectively, were performed to measure *HtrA4* mRNA (**E**) and HtrA4 protein (**F**) in BeWo WT and BeWo KO cells. Each experiment was performed in triplicate.

Analysis of the tissue-profiling microarray data revealed that *HtrA4* mRNA was primarily expressed in the human placenta compared to other human tissues [12]. Singh et al. reported that *HtrA4* mRNA was highly expressed in the placental tissue and the BeWo cell line (placental cell line), and mildly expressed in the breast tissue [12]. Our western blot analysis indicated that HtrA4 expression in BeWo cells was higher than the levels obtained from JEG3 and HTR-8/SVneo cell lines (Figure S2A). To investigate the functions of HtrA4 in trophoblast cells, we generated a knockout of *HtrA4* in the BeWo cell line using the CRISPR/Cas9 system. When the cells reached exponential growth (70~80% confluence), we transfected sgRNA with electroporation, after which almost half of the cells died due to the effect of electroporation on BeWo cells. We obtained a transfection efficiency of 20~30% of the px458-sgRNA plasmid into BeWo cells (Figure 1A).

Moreover, we designed primers for PCR to obtain sgRNA-targeting gene fragments from two transfected monoclonal BeWo cells. We detected a single band in the knockout cell line 1, and two bands in the knockout cell line 2 (Figure 1B). Subsequently, we performed the T7E1 assay for PCR products (Figure 1C), which revealed that the *HtrA4* gene in both independent monoclonal BeWo cell lines was knocked out, and both *HtrA4* alleles in knockout cell line 2 were knocked out since four bands were detected (Figure 1C lines 5 and 6). We examined two PCR products from the T7E1 assay for their base sequence after TA cloning. Direct sequencing revealed a deletion of 95 bases in allele 1, and 35 bases in allele 2 (Figure 1D). These deleted bases were not in multiples of 3, indicating that the base sequences in the *HtrA4* gene were disrupted, resulting in a translated protein that was not intact HtrA4. As expected, *HtrA4* mRNA was barely detectable using RT-PCR (Figure 1E), and no HtrA4 was detected in the western blot analysis of the BeWo KO cells (Figure 1F). We investigated a likely potential off-target site using the ‘Cas-OFFinder’ bioinformatics tools website (Figure S2B). The off-target site belongs to an intron (https://genome.ucsc.edu/cgi-bin/ accessed on 3 April 2023) that does not transcribe mRNA and translates into a protein (Figure S2C); hence, we conducted no further experiments to examine the knockout for off-target sites. To check whether the off-target is one of the HtrA family members (including *HtrA1*, *HtrA2*, and *HtrA3*), we performed alignment analysis. Our results showed seven mismatched sequences in *HtrA1* and *HtrA3*, when compared to the designed sgRNA targeted genes, whereas five target sequences were found to be mismatched in *HtrA2* (Figure S2D).

### 3.2. Effect of HtrA4 on Cell Fusion

**Figure 2:**
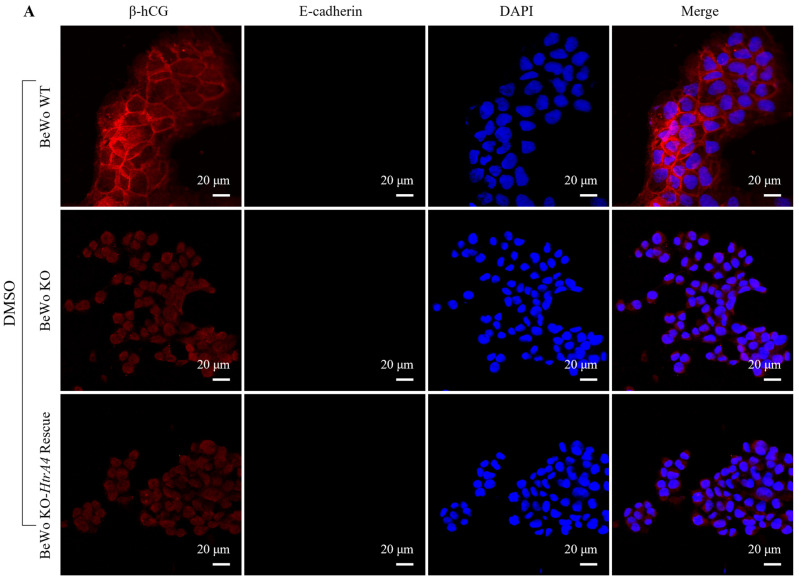

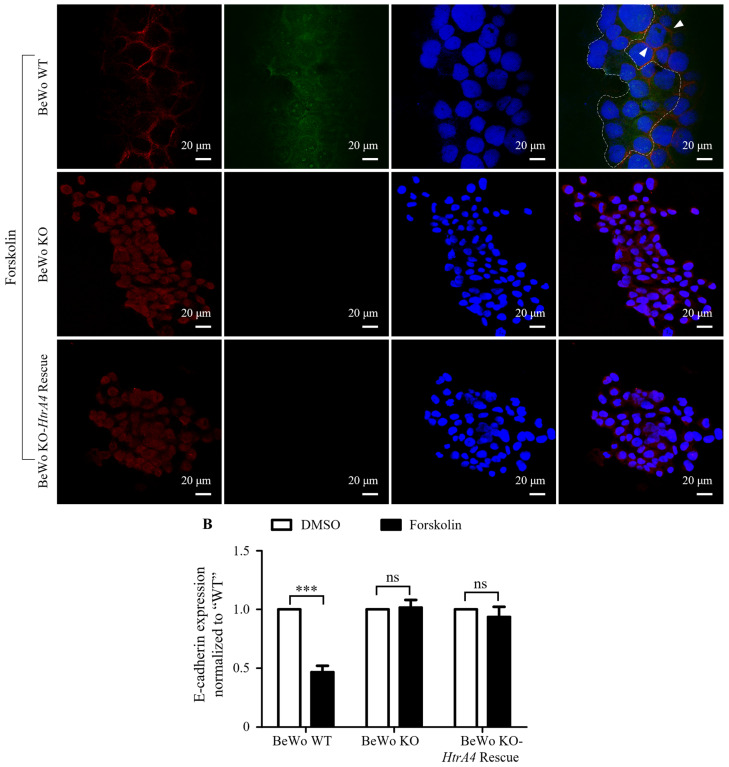
Effect of HtrA4 on cell fusion. (**A**) BeWo WT, BeWo KO, and BeWo KO-*HtrA4* rescue cells were subjected to two conditions: they were treated with 50 μM of forskolin or equal amounts of DMSO to induce cell fusion. After 48 h, an immunocytochemistry analysis was performed to examine the expression of E-cadherin and β-hCG. nuclei were stained blue with DAPI, while E-cadherin and β-hCG were labelled with red and green, respectively. (**B**) Quantification of the normalized fluorescence intensity of E-cadherin. *** *p* < 0.001, ns *p* > 0.05. Each experiment was performed in triplicate.

The BeWo cell line is known to effectively syncytialize in culture when treated with forskolin or cAMP [25]. To investigate the role of HtrA4 in BeWo cell fusion, BeWo WT, BeWo KO, and BeWo KO-*HtrA4* rescue cells (Figure S2A–C) were exposed to forskolin or an equal amount of DMSO for 48 h, and the expression of E-cadherin and β-hCG was analyzed via immunocytochemistry. The results revealed an interesting phenomenon: the cell–cell fusion did not occur in BeWo KO and BeWo KO-*HtrA4* rescue cells, and the expression of β-hCG was not detected in those cells. Notably, although the expression of E-cadherin still exists in BeWo KO and BeWo KO-*HtrA4* rescue cells, these cell types do not form linear cell–cell junction structures compared to BeWo WT cells. Additionally, in BeWo WT cells treated with DMSO, E-cadherin forms a clearly linear cell–cell junction structure. However, in BeWo WT cells undergoing cell fusion after forskolin treatment, E-cadherin does not form or forms discontinuous linear cell–cell junction structures (Figure 2A,B), and there is a gain of β-hCG expression (Figure 2A). These findings suggest that HtrA4 is crucial for BeWo cell syncytialization and plays a role in the secretion of β-hCG by trophoblasts.

### 3.3. Effect of HtrA4 on Cell Invasion and Migration

**Figure 3:**
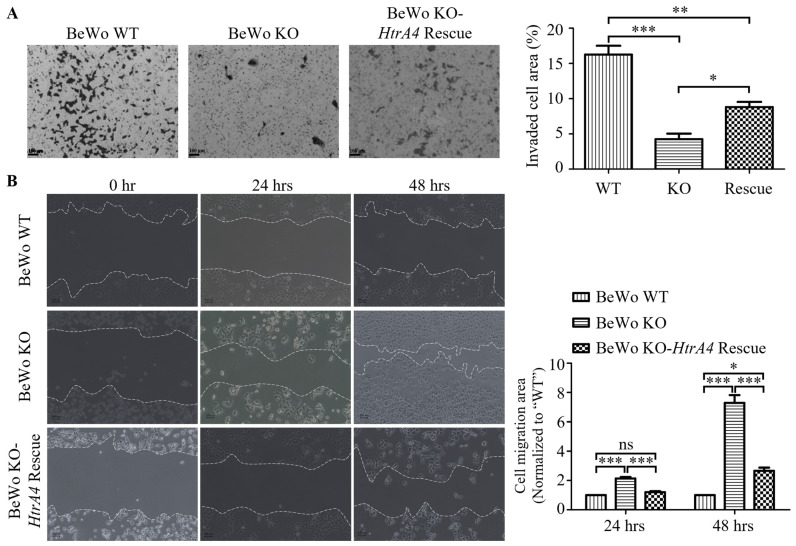

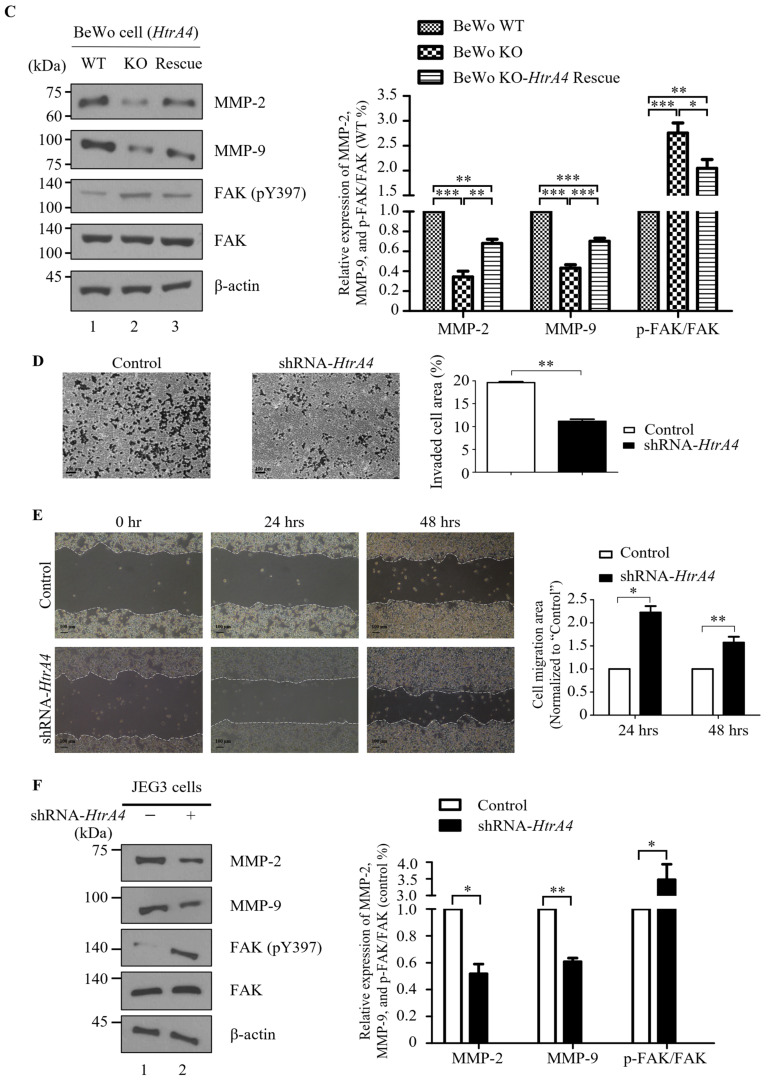
Transwell invasion and scratch wound healing assays in BeWo and JEG3 cells. (**A**) Representative images of the invasion assay in BeWo WT, BeWo KO, and BeWo KO-*HtrA4* rescue cells were obtained, and cell counts were compared among the groups. (**B**) Representative images of the wound healing assay in BeWo WT, BeWo KO, and BeWo KO-*HtrA4* rescue cells were obtained after treatment with mitomycin C, and migration distances were compared. (**C**) Western blot analysis was performed with 30 μg of protein from whole cell lysate to determine the expressions of MMP-2, MMP-9, p-FAK (pY397), and FAK. (**D**) Representative images of the invasion assay in JEG3 control and shRNA-*HtrA4* JEG3 cells were obtained, and cell counts were compared between the groups. (**E**) Representative images of the wound healing assay in JEG3 control and shRNA-*HtrA4* JEG3 cells were obtained, and migration distances were compared. (**F**) Western blot analysis was performed with 30 μg of protein from whole cell lysate to determine the expressions of MMP-2, MMP-9, p-FAK (pY397), and FAK. β-actin was used as an internal control, and the statistical analysis of protein quantification using western blotting was performed using *t*-test. Data are presented as the means ± standard error (SD). * *p* < 0.05, ** *p* < 0.01, *** *p* < 0.001, ns *p* > 0.05. Each experiment was performed in triplicate.

BeWo KO cells showed a significant reduction in invasion activity compared to the BeWo WT cells. However, when *HtrA4* was rescued in BeWo KO cells, the invasion ability increased (WT vs. KO *p* < 0.001, WT vs. rescue *p* < 0.01, KO vs. rescue *p* < 0.05) (Figure 3A). To investigate the role of HtrA4 in cell migration, a scratch wound assay was performed. Interestingly, the migration ability of BeWo WT and BeWo KO-*HtrA4* rescue cells was significantly lower than BeWo KO cells after treatment with mitomycin (cell migration distance in 24 h: WT vs. KO *p* < 0.001, rescue vs. KO *p* < 0.001; cell migration distance in 48 h: WT vs. KO *p* < 0.001, rescue vs. KO *p* < 0.001, WT vs. rescue *p* < 0.05) (Figure 3B). Previous studies have consistently demonstrated the role of matrix metalloproteinase 2 (MMP-2) and matrix metalloproteinase 9 (MMP-9) in promoting trophoblast cell invasion [26,27]. Additionally, focal adhesion kinase (FAK) has been implicated in trophoblast cell migration [28,29]. In this study, our aim was to investigate whether HtrA4 modulates the signaling pathways associated with MMP-2, MMP-9, and FAK in relation to cell invasion and migration. We performed western blot analysis to assess the expression levels of MMP-2, MMP-9, p-FAK (Y397), and FAK in BeWo and JEG3 cells. Our results revealed that the knockout of *HtrA4* significantly decreased the expression levels of MMP-2 and MMP-9, while the ratio of p-FAK (pY397)/FAK was significantly higher in BeWo KO compared to BeWo WT cells (Figure 3C). Interestingly, when we reintroduced *HtrA4*-Flag expression in BeWo KO cells, we observed an increase in MMP-2 and MMP-9 expression, accompanied by a decrease in the p-FAK (pY397)/FAK ratio (Figure 3C). Moreover, we found that downregulating the expression of HtrA4 (Figure S3B) significantly impaired the invasion ability (control vs. shRNA-*HtrA4 p* < 0.01) (Figure 3D), while enhancing the migration ability (cell migration distance in 24 h: control vs. shRNA-*HtrA4 p* < 0.05; cell migration distance in 48 h: control vs. shRNA-*HtrA4 p* < 0.01) (Figure 3E). Furthermore, the invasion-related factors MMP-2 (control vs. shRNA-*HtrA4* JEG3 *p* < 0.05) and MMP-9 (control vs. shRNA-*HtrA4* JEG3 *p* < 0.01) were reduced in shRNA-*HtrA4* JEG3 cells, while the migration-related factor p-FAK (pY397)/FAK (control vs. shRNA-*HtrA4* JEG3 *p* < 0.05) (Figure 3F) was increased. These findings suggest that HtrA4 promotes the invasion of BeWo and JEG3 cells by modulating the levels of MMP-2 and MMP-9 and downregulating the phosphorylation of FAK at tyrosine 397 (pY397), resulting in the inhibition of migration in BeWo and JEG3 cells.

### 3.4. Effect of HtrA4 on Cell Proliferation

**Figure 4:**
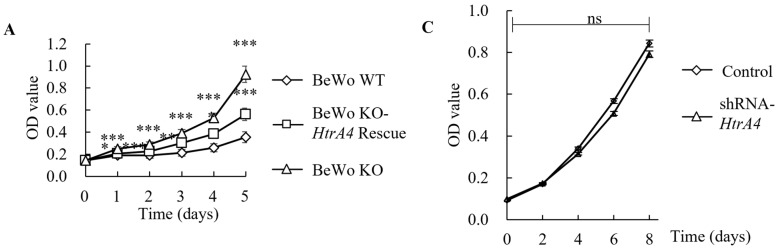

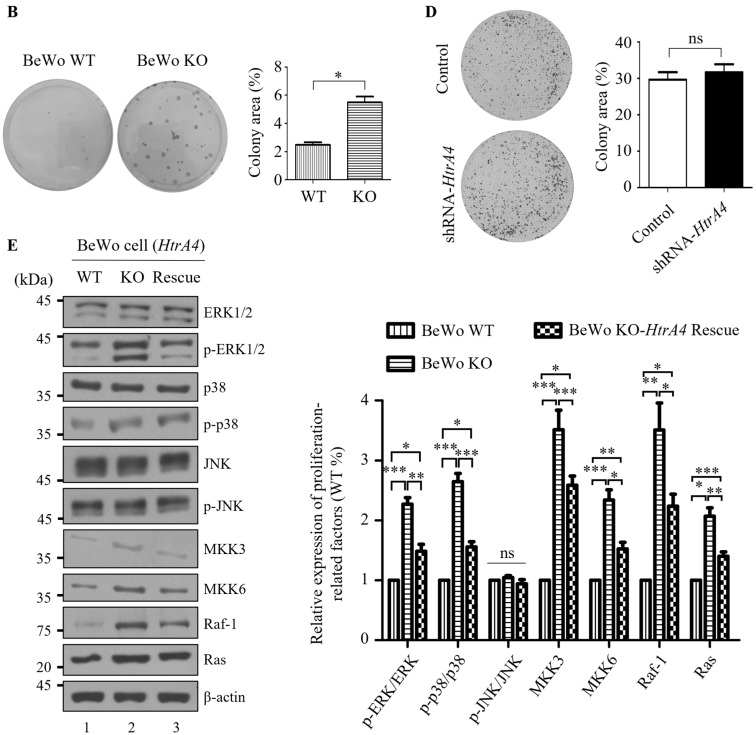
Effect of HtrA4 on cell proliferation. (**A**) Cell viability was evaluated using CCK-8 assay in BeWo WT, BeWo KO, and BeWo KO-*HtrA4* rescue cells, and optical density (OD) values were measured at 0, 1, 2, 3, 4, and 5 days. (**B**) The colony formation assay was conducted in BeWo WT and BeWo KO cells, and representative images were captured. The number of cell colonies was compared between the two groups. (**C**) CCK-8 assay was conducted in control and shRNA-*HtrA4* JEG3 cells, and OD values were measured at 0, 2, 4, 6, and 8 days. (**D**) The colony formation assay was conducted in JEG3 cells, and representative images were captured. The number of cell colonies was compared between control and shRNA-*HtrA4* JEG3 cells. (**E**) Western blot analysis was conducted using 30 μg of proteins from whole cell lysate to determine the expressions of ERK1/2, p-ERK1/2, p38, p-p38, JNK, p-JNK, Ras, Raf-1, MKK3, and MKK6. β-actin was used as an internal control. Statistical analysis of protein quantification in western blotting was performed using a *t*-test. Data are presented as the means ± standard error (SD). * *p* < 0.05, ** *p* < 0.01, *** *p* < 0.001, ns *p* > 0.05. Each experiment was conducted in triplicate.

We conducted CCK-8 and CFU assays to investigate the effect of HtrA4 on cell proliferation. The assays revealed that BeWo KO cells had a significantly higher proliferative capacity than BeWo WT cells (Figure 4A,B). However, when HtrA4 was reintroduced into BeWo KO cells, the proliferative capacity decreased. Moreover, transfecting shRNA-*HtrA4* into JEG3 cells increased their proliferative capacity, but there was no significant difference between the control and shRNA-*HtrA4* JEG3 cells (Figure 4C,D). Furthermore, western blot analysis revealed that in BeWo KO cells, the expression levels of factors associated with cell proliferation were the highest, including extracellular-signal-regulated kinase (p-ERK1/2/ERK1/2), p-p38/p38, Ras, rapidly accelerated fibrosarcoma-1 (Raf-1), mitogen-activated protein kinase 3 (MKK3), and mitogen-activated protein kinase 6 (MKK6). This was followed by BeWo KO-*HtrA4* rescue and BeWo WT cells. However, there was no significant difference in Jun N-terminal kinase (p-JNK/JNK) expression between BeWo KO and BeWo WT cells (Figure 4E).

### 3.5. Effect of HtrA4 on Cell Cycle

**Figure 5:**
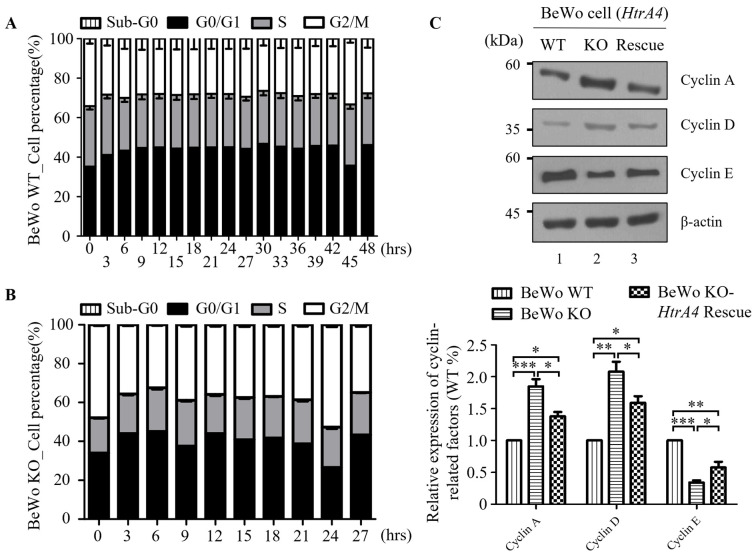
Effect of HtrA4 on cell cycle. (**A**) Cell cycle distribution and quantitative measurement of cell cycle phases in BeWo WT cells over a 3 h period. (**B**) Cell cycle distribution and quantitative measurement of cell cycle phases in BeWo KO cells over a 3 h period. (**C**) Western blot analysis was performed with 30 μg of proteins to determine the expression levels of Cyclin D, Cyclin A, and Cyclin E. β-actin was used as an internal control. Statistical analysis of protein quantification in western blotting was performed using a *t*-test. Data are presented as the means ± standard error (SD). * *p* < 0.05, ** *p* < 0.01, *** *p* < 0.001. Each experiment was performed in triplicate.

Cell proliferation in BeWo cells was examined in Section 3.4. To investigate whether BeWo KO cells exhibit a higher proliferation rate than BeWo WT cells, we conducted flow cytometry analysis to determine the cell cycle of each cell line. Notably, the cell cycle of BeWo WT cells was approximately 45 h (Figure 5A and Figure S4A), whereas the cell cycle of BeWo KO cells was significantly shorter, at approximately 24 h (Figure 5B and Figure S4B). When BeWo WT cells were cultured, the proportion of cells in the G0/G1 phase was 27.47% at 0 h, while the proportion of cells in the G2/M phase was 40.95%. This pattern of cell cycle distribution was consistent throughout the 45 h culture period, with no significant changes observed between 3 and 42 h of culture. Therefore, we infer that the cell cycle duration for BeWo WT cells is approximately 45 ± 3 h, while that of BeWo KO cells is approximately 24 ± 3 h. Additionally, we conducted western blot analysis to assess the expression levels of Cyclins, A, D, and E. As depicted in Figure 5C, the maximum levels of Cyclin A and Cyclin D, and the minimum level of Cyclin E, were observed in BeWo KO cells. Additionally, the reintroduction of *HtrA4*-Flag led to a decrease in the expression of Cyclin A and Cyclin D, or an increase in the expression of Cyclin E in BeWo KO cells.

### 3.6. HtrA4 Expression Is Lower in Sera of RPL Patients

**Figure 6:**
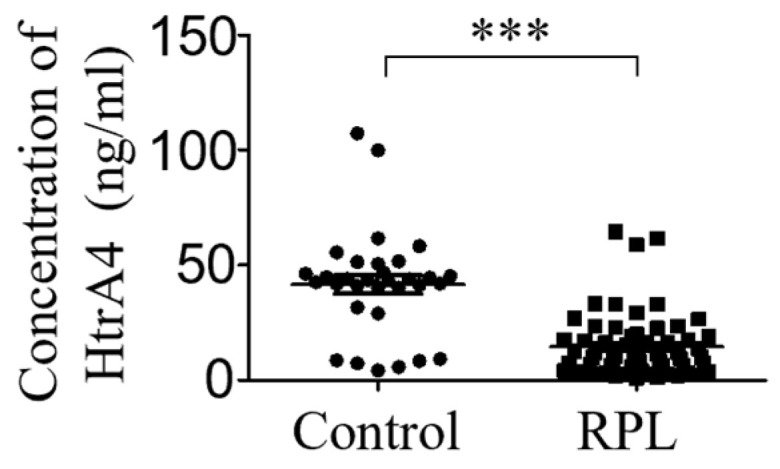
Detection of serum HtrA4 level. The HtrA4 protein was determined using ELISA in sera obtained from normal controls and RPL patients with regular menstrual cycles on the 5th to 9th days after ovulation in the menstrual cycle. *** *p* < 0.001. The experiment was performed for 32 controls and 60 RPL patients at the same time.

Previous studies have shown that *HtrA4* mRNA expression is reduced in the villi of patients with RPL [19]. While HtrA4 has potential as a biomarker for diagnosing RPL, villus sampling during pregnancy is an invasive procedure. Therefore, we aimed to investigate the changes in serum HtrA4 levels after abortion and evaluate their correlation with non-pregnancy status using the HtrA4 ELISA kit. As shown in Figure 6, the serum HtrA4 concentration in RPL patients was found to be significantly lower than that in the control group (serum HtrA4 concentration: control: 41.68 ± 4.054 ng/mL, RPL: 14.56 ± 1.806 ng/mL, *p* < 0.001).

## 4. Discussion

The development and function of the placenta are closely related to the fusion, proliferation, invasion, and migration functions of trophoblast cells [30]. Among several trophoblast cell lines, the BeWo cell line has been shown to have a high expression of HtrA4 [12]. Additionally, when stimulated with forskolin or cAMP, BeWo cells are the only cell line that can successfully syncytialize in culture [31,32]. To explore the functions of HtrA4 in cell fusion, invasion, migration, proliferation, and cell cycle, we performed *HtrA4* knockout experiments in BeWo cells and *HtrA4* knockdown experiments in JEG3 cells. Our data indicate that HtrA4 plays a critical role in the cellular functions of both BeWo and JEG3 cells.

Mansilla et al. reported that HtrA4 expression is upregulated after the spontaneous syncytialization of primary trophoblasts from human placenta or the forskolin-induced syncytialization of BeWo cells [25]. Furthermore, they demonstrated that the upregulation and secretion of β-hCG, as well as the concomitant downregulation of E-cadherin, indicate effective syncytialization in BeWo WT cells, whereas such regulation of β-hCG and E-cadherin is not observed in BeWo KO cells [25]. Our data also revealed the upregulation of β-hCG and the downregulation of E-cadherin in the forskolin-induced syncytialization of BeWo WT cells. In contrast, the absence of *HtrA4* results in the prevention of cell fusion and β-hCG secretion, and the reintroduction of *HtrA4* did not restore cell–cell fusion. These observations may be attributed to the disruption of cell-to-cell junction structures. Further investigation is required to elucidate the mechanism by which *HtrA4* knockout affects E-cadherin. When the effect of HtrA4 on cell fusion in HEK293T cells was investigated, the results were reversed compared to those in BeWo cells [9]. Wang et al. reported that HtrA4 suppresses cell–cell fusion by inhibiting the activity of syncytin-1 in HEK293T cells [9]. This may be related to the fact that HEK293T cells are derived from a human embryonic kidney cell line, rather than a trophoblast cell line, and they do not naturally syncytialize or produce and secrete hormones or enzymes such as β-hCG and HtrA4. Our findings suggest that HtrA4 is an essential factor in the syncytial process of BeWo cells and is closely related to the production and secretion of β-hCG during forskolin-induced cell–cell fusion.

The invasive and migratory cytotrophoblasts are crucial in the modeling of uterine spiral artery [26]. Several studies suggest that HtrA4 is involved in cell invasion and migration. In previous studies, Wang et al. demonstrated that GCM1 overexpression promotes cell invasion in JAR (trophoblast cell line) and BeWo cells by upregulating the expression of HtrA4, which facilitates extracellular matrix (ECM) cleavage [9]. Conversely, Chiu et al. reported that GATA3, an upstream factor of GCM1, negatively regulates GCM1′s transcriptional activity, resulting in the suppression of HtrA4 expression [11]. Knocking down *GATA3* was found to enhance cell invasion in JEG3 and BeWo cells [11]. Additionally, Wang et al. found that knockdown of *RACK1* downregulates GCM1 stability, leading to the inhibition of HtrA4 expression, thereby suppressing cell invasion and migration in BeWo cells [10]. These findings suggest that the upregulation of HtrA4 promotes cell invasion, while the downregulation of HtrA4 inhibits cell invasion. Consistent with these observations, our study demonstrates that knocking out *HtrA4* in BeWo cells or reducing *HtrA4* expression in JEG3 cells significantly impairs cell invasion capacity (Figure 3A,D).

However, our findings differ from those of Wang et al. regarding cell migration. Our results indicate that knocking out *HtrA4* in BeWo cells or reducing *HtrA4* expression in JEG3 cells enhances cell migration capacity (Figure 3B,E). In contrast, Wang et al. showed that reducing RACK1 expression in BeWo cells decreases GCM1 stability, thereby inhibiting HtrA4 expression and suppressing cell migration [10]. This discrepancy in the role of HtrA4 in cell migration may be attributed to the different ways of regulating HtrA4 expression. In our study, *HtrA4* expression was manipulated directly using sgRNA or shRNA-*HtrA4*, while Wang et al. modulated HtrA4 expression by controlling GCM1 stability through RACK1. It is noteworthy that RACK1 is known to regulate cell migration through various signaling pathways, such as the modulation of Src kinase activity, the regulation of cell polarity and direction sensing, and the promotion of the epithelial to mesenchymal transition [33]. However, in non-small cell lung cancer, RACK1 mediates the assembly of the RACK1–PP2A–Akt signaling complex in response to EphB3 activation, leading to decreased Akt phosphorylation and the inhibition of cell migration [34]. Since RACK1 can regulate the expression of multiple proteins when overexpressed or knocked down [33], the effect of reduced HtrA4 on cell migration in our study might be masked by other signaling pathways. Additionally, the findings of Wenta et al. align with the trends observed in our study. They demonstrated that cancerous A549 and MCF7 cells with repressed *HtrA4* gene expression through shRNA exhibited enhanced cell migration compared to control cells, while A549 cells with exogenous HtrA4 production displayed decreased migration efficiency [4].

On the contrary, proteins generally promote both cell invasion and cell migration in most cases. However, our experimental results demonstrate the inconsistent effects of HtrA4 on BeWo cell phenotypes. The reduced expression of HtrA4 inhibits cell invasion but promotes cell migration. We believe that this inconsistency may be attributed to the specific characteristics of the HtrA4 protein and the signaling pathways involved. Wang et al. showed that the overexpression of HtrA4 in JEG3 [9] and JAR [35] cells promotes cell invasion, whereas overexpression in HTR8/SVneo cells inhibits cell migration [13]. Additionally, Zhang et al. reported similar findings, revealing that cyclic mechanical stretching enhances the migration of bone marrow stromal cells (BMSCs) while suppressing cell invasion [36]. They demonstrated that cyclic mechanical stretching promotes BMSC migration by upregulating p-FAK and p-ERK levels, and it inhibits BMSC invasion by reducing MMP-2 and MMP-9 secretion via the upregulated p-FAK, which is independent of the ERK signaling pathway [36]. Our results also demonstrate increased levels of p-FAK and p-ERK in BeWo KO cells, along with decreased levels of MMP-2 and MMP-9. Cell invasion typically involves the process of tumor progression and focuses on the invasion of cells into the extracellular matrix (ECM) [37]. MMP-2 and MMP-9 are involved in the degradation of ECM components during cell invasion [27,29,38]. FAK is generally known to promote cell invasion, migration, and increase MMP production [39]. Several studies suggest that FAK can promote the secretion of MMP-2 and MMP-9 through the ERK signaling pathway [40,41]. Furthermore, several downstream molecules of FAK, such as RhoA, JNK1/2, and Rac1, have been shown to regulate the expression of MMP-2 and MMP-9 [42]. Conversely, cell migration refers to the movement of cells themselves, and the mechanical properties of actively migrating cells are believed to be determined by the cytoskeletal network [43]. The FAK/Src/Cas/Rho GTPase signaling pathway controls cell migration by modulating the cytoskeleton [44]. The coordinated assembly and breakdown of actin filaments provide the primary energy source for cell migration, and the Rho family GTPases Cdc42, Rac1, and RhoA are essential components of this process [45]. Different populations of actin filaments and their related adhesion are regulated by the activity of Rac1, Cdc42, and RhoA [45]. Rac1 activation facilitates the formation of precursor adhesions [46], while Cdc42 stimulation leads to the development of filopodia with parallel-arranged actin filament bundles [47]. RhoA activation is associated with the formation of actin stress fibers and mature focal adhesions [48]. Therefore, we speculate that these signaling molecules may be involved in the mechanisms by which cell invasion is inhibited and cell migration is promoted upon the reduction of HtrA4. However, further research is required to confirm this. Based on our study results, we propose that p-FAK (Y397) may be involved in different signaling pathways to promote cell migration and inhibit cell invasion in BeWo and JEG3 cells. These findings further emphasize the critical role of HtrA4 in regulating cell invasion and migration.

The normal trophoblast turnover is maintained through cell proliferation and apoptosis, which play a crucial role [22]. In this study, our findings demonstrate that *HtrA4* knockout promotes the BeWo cell proliferation by activating the Ras-Raf-ERK and MKK3/MKK6-p38 signaling pathways, but not the JNK signaling pathway. Previous research has shown that cell proliferation is linked to the ERK, p38, and JNK signaling pathways [49]. However, the capacity for cell proliferation was increased in JEG3 cells with *HtrA4* knockdown, although the increase was not statistically significant, possibly due to the transfected status of JEG3 cells or the residual presence of HtrA4. Schmidt et al. also observed similar results, where gene knockout or knockdown promoted cell proliferation, but the increase was not statistically significant in the gene knockdown. For instance, knocking out the *HtrA1* gene in the mouse embryonic fibroblasts (MEF) cells accelerated proliferation, but downregulating *HtrA1* in SW480 cells via shRNA-*HtrA1* did not significantly affect cell proliferation [50]. Upon further examination, we observed that the morphology of BeWo cells changed after knocking out the *HtrA4* gene, with a significant reduction in the size of the nuclei compared to wild-type cells. After rescuing HtrA4, we observed that the size of the cell nucleus remains unchanged. This finding suggests that the restored HtrA4 may have been ineffective in modifying the signaling pathways responsible for regulating the cell nucleus size. However, to draw a definitive conclusion, further confirmation is required. Similar findings were reported by Schmindt et al. for MEF cells when the *HtrA1* gene was knocked out [50]. Additionally, research has shown that an increase in cell proliferation rates is often associated with a decrease in cell size and volume [50]. Thus, we propose that the promotion of cell proliferation resulting from the knockout of *HtrA4* and *HtrA1* genes may be attributed to this mechanism. Emerging evidence suggests that cell proliferation also depends on cell cycle signals, and each cell line has its inherent cell cycle [51]. However, the function of HtrA4 in the BeWo cell cycle is poorly studied. Wang et al. reported that HtrA4 inhibits the proliferation of HUVECs and endothelial progenitor cells (EPCs) [52]. The RT2 Profiler PCR array data revealed that the expression of 35 genes (G1 phase and S phase-, G2 phase and M phase-, cell cycle checkpoint and cell cycle arrest-, and regulation of the cell cycle-related genes) were downregulated by 2-fold in HUVECs treated with a high level of recombinant HtrA4 [52]. Interestingly, the high level of HtrA4 had no effect on apoptosis-related genes (*Casp3* and *Bcl2*). These results suggest that the high level of HtrA4 inhibits cell proliferation due to the downregulation of cell cycle-related gene expression and not the induction of cell death. Cyclins play different roles in each phase of the cell cycle. Cylin D interacts with and activates CDK4/CDK6, forming the Cyclin D-CDK4/6 complex to promote G1 phase progression [53]. As Cyclin E levels fall, Cyclin A levels rise, and it is considered that CDK2/Cylin A activity propels cells through the S phase [54]. Additionally, Cyclin A forms a complex with CDK1, which stabilizes and activates the Cyclin B/CDK1 complex for the G2 phase [55,56]. Our data demonstrate that the depletion of HtrA4 upregulates Cyclin D and Cyclin A levels and downregulates Cyclin E levels (Figure 5C). Based on these findings, we infer that the upregulated levels of Cyclin D and Cyclin A may promote G1 and G2 phase progression to shorten the cell cycle, which can lead to anarchic proliferation in BeWo KO cells. 

Due to the unpredictable nature of miscarriages and patient mobility, collecting blood samples from RPL patients at the time of miscarriage is challenging. As an alternative, we screened blood samples from outpatients previously diagnosed with RPL. In a previous report, we found lower *HtrA4* mRNA expression in the chorionic villi of RPL patients compared to normal controls. Our ELISA results (Figure 6) are consistent with this finding. However, these results alone do not fully demonstrate the potential of low serum concentration as a biomarker for RPL. Instead, they serve as a reference for predicting the risk of miscarriage in subsequent pregnancies for patients with RPL. To investigate the correlation between serum HtrA4 levels and RPL in pregnant and non-pregnant states, a large sample cohort study is required. If confirmed that reduced HtrA4 is detectable in serum or placenta, HtrA4 could serve as a potential biomarker for RPL and a therapeutic target for drug development. One limitation of this study is the use of BeWo and JEG3 cells as primary trophoblast cell models, which could be improved by utilizing isolated primary trophoblasts to highlight the importance of HtrA4 functions in placenta.

## 5. Conclusions

The data obtained from BeWo and JEG3 cells indicate that reduced HtrA4 expression may lead to abnormal physiological effects on trophoblasts, including decreased cell fusion and invasion, increased migration and proliferation, and a shortened cell cycle, potentially affecting placental functions.

## Figures and Tables

**Table 1:** The general characteristics and past history of study participants, including normal controls and patients with RPL, were examined.

Characteristics		Control (*n* = 32)	RPL (*n* = 60)	*p*-Value
Age (y)		31.8 ± 4.4	32.6 ± 5.2	ns
BMI		24.9 ± 2.4	26.3 ± 1.7	ns
Gravidity (*n*)		2.5 ± 1.6	5.3 ± 2.6	<0.01 **
Parity (*n*)		1.8 ± 0.5	1.4 ± 0.2	<0.05 *
Previous pregnancy losses (wk)		-	7.8 ± 1.5	-
Spontaneous abortion (no.)		0 ± 0	3.6 ± 0.8	-
Artificial abortion (no.)				
times	0	20 (62.5%)	0	
	1~2	12 (37.5%)	0	

Note: Data were presented as an *n* or means ± SD; * *p* < 0.05, ** *p* < 0.01, ns: not significant. Abbreviation: BMI, Body mass index.

## Data Availability

Written informed consent has been obtained from the patient(s) to publish this paper.

## Supplementary Materials

The following supporting information can be downloaded at: https://www.mdpi.com/article/10.3390/cells12111459/s1, Figure S1. Schematic diagram of the *HtrA4*-sgRNA-pX458 expression construct. Figure S2. Establishment of *HtrA4* knockout cell line. Figure S3. Expression of HtrA4 in BeWo and JEG3 cells. Figure S4. Cell cycle distribution of BeWo cells at 3 h. Table S1. Information of designed sgRNA primers. Table S2. Information of primers for PCR and RT-PCR. Table S3. Antibodies information.
