# Supplementary material for: Cellular Functions of High-Temperature Requirement Factor A4 in Placenta
Source: Cells. 2023 May 24;12(11):1459. doi: 10.3390/cells12111459 (PMC10252923; doi:10.3390/cells12111459)
Supplement: Supplementary file 1 [file cells-12-01459-s001.zip › cells-2365688-supplementary.pdf]

Supplementary Materials

1 Supplementary Figures and Tables

1.1 Supplementary Figure S1

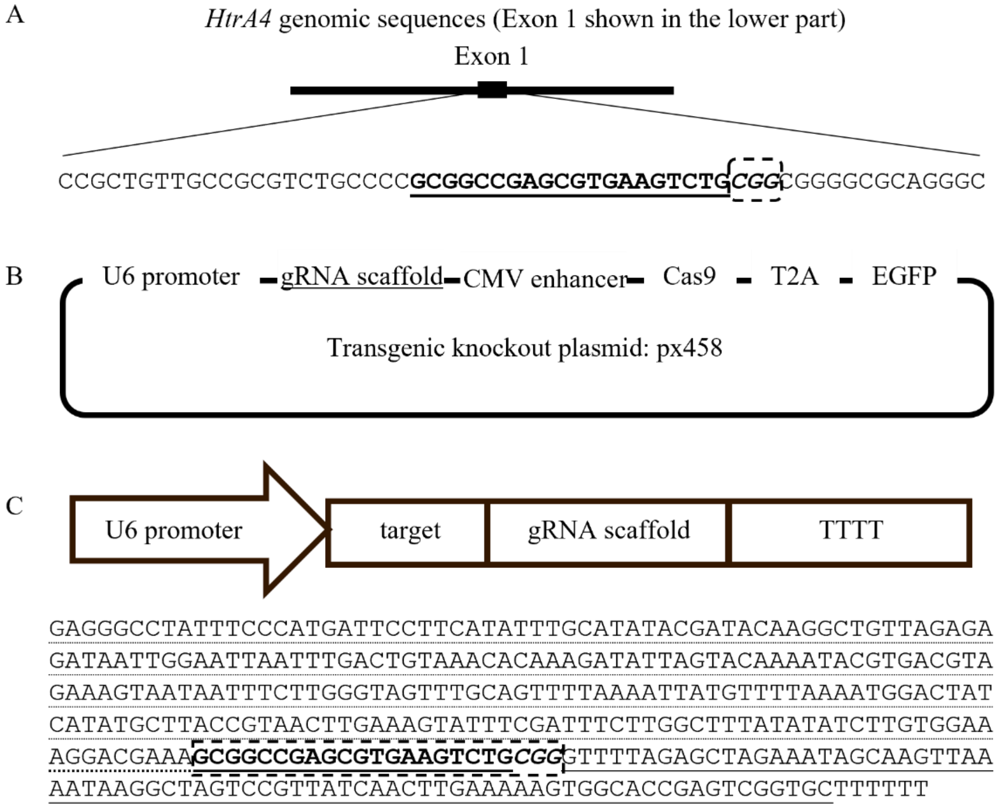

Supplementary Figure S1. Schematic diagram of the pX458-sgRNA-*HtrA4* expression construct. (A) The *HtrA4* cleavage site is depicted in its genomic context, with the target guide RNA (gRNA) sequence for *HtrA4* indicated by an underline. (B) The pX458-sgRNA-*HtrA4* expression plasmid contains four genes: gRNA scffold, CMV enhancer, Cas9, and EGFP. (C) The gRNA is expressed using the U6 promoter-based expression scheme.

1.2 Supplementary Figure S2

A

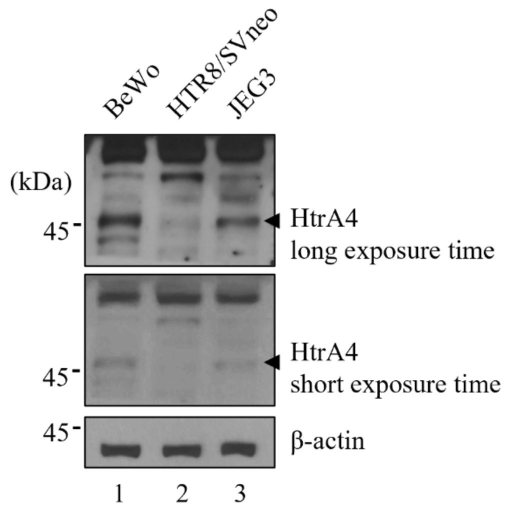

B

Bulge type	Target	Chr	Position	Direction	Mismatches	Bulge size
X	crRNA: GCGGCCGAGCGTGAAGTCTGNGG<br>DNA: GCGGCCGAGCGTGAAGTCTGCGG	Chr 8	38974482	+	0	0
X	crRNA: GCGGCCGAGCGTGAAGTCTGNGG<br>DNA: GCaGCtGAGCcTGAAGTCTGAGG	Chr 6	30890735	+	3	0

C

Off target	Sequence	UCSC gene	Locus
Off target 1	GCAGCTGAGCCTGAAGTCTGAGG	Intron	30890735-30890758

D

```
HtrA2   --------------------------------------ATGGCTGCGCCGAGGGCGGGGC 22
HtrA4   ATGATTAGACCTCAGCTGCGGACCGCGGGGCTGGACGATGCCTCCTGCCGGGGCTGCTG 60
HtrA1   ---------------ATGCAGATCCCGCG------CGCCGCTCTTCTCCCGCTGCTGCTG 39
HtrA3   ---------------------------------------------ATGCAGGCGCGAGCG 15
HtrA2   GGGGTGCAGGCTGGAGCCTTCGGGCATGGCGGGCTTTGGG-GGGCA---TTCGCTGGGGG 78
HtrA4   CTGCTCCTGG----TGCCCGTCCTCTGGCCGGGGCTGAA---AAG---------CTACA 104
HtrA1   CTGCTGCTGG----CGGCGCCCGCCTCGGCGCAGCTGTCCCGGGCCGGCCGCTCGGCGCC 95
HtrA3   CTGCTCCTGG----CCGCGTTGGCCGCGCTGGCGCTGGCCCGGGAGCCCCCTGCGGCGC- 70
HtrA2   AGGAGACCCCGTTTGACCCCTGACCTCCGGGCCCTGCTGACGTCAGGAACTTCTGACCCC 138
HtrA4   TACCCAGCCCTCCTGCCCCGCGGTCTGCCAGCCCACGCGCTGCCCCGCGCTGCCCACCTG 164
HtrA1   TTTGGCCGCCGGGTGCCCAGACCGCTGCGAGCCGGCGCGCTGCCCGCCGCAGCCGGAGCA 155
HtrA3   -----------CGTGTCCCGCGCGCTGCGACGTGTCGCGGTGTCCCAGC------CCCCG 113
HtrA2   CGGGCCCGAGTGACTTATGGACCCCCAGTCTCTGGGCCCGGTTGTCTGTTGGGGTCACT 198
HtrA4   CGCGCT----------GGGGACCACGCCGGTGTTCGACCTGTGCCGCTGTTGCCGCGTCT 214
HtrA1   CTG-------------CGAGGGCGGCCGGGCCCGGGACGCGTGCGGCTGCTGCGAGGTGT 202
HtrA3   CTG-------------CCCCGGCGGCTACGTGCCCGACCTCTGCAACTGCTGCCTGGTGT 160
HtrA2   GAACCGCGAGCATGCCTGACGTCTGGGACCC----------------CGGGTCCCCGGGC 242
HtrA4   GCCCCGCGGCCGAGCGTGAAGTCTGCGGCGGGGCGCAGGGCCAACCGTGCGCCCCGGGGC 274
HtrA1   GCGGCCCGCCCGAGGGCGCCGCGTGCGGCCT---GCAGGAGGGCCCGTGCGGCGAGGGGC 259
HtrA3   GCGCCCCCACCGAGGGCGAGCCGTGTGGCGGCCCTCTGGACTCGCCTTGCGGCGAGAGCC 220
        *    * *   *  *   *     ** * *                   * * *    * *
```

Supplementary Figure S2. Establishment of *HtrA4* knockout cell line. (A) Expression of HtrA4 proteins was investigated in the BeWo WT, HTR8/SVneo, and JEG3 cell lines by western blotting

analysis. (B) Off-target analysis of the sgRNA used for BeWo cell correction. The single off target site was identified using "Cas-OFFinder" software (http://www.rgenome.net/cas-offinder/); (C) Gene ID and genomic locus information were obtained from the "Genome Browser Gateway" website (https://genome.ucsc.edu/cgi-bin/hgGateway). (D) Alignment analysis was performed with *HtrA1*, *HtrA2*, *HtrA3*, and *HtrA4* sequences. The sgRNA target sites are marked with a yellow underline, the PAM sequence with a green underline, and the matched sequences in *HtrA1*, *HtrA2*, *HtrA3*, and *HtrA4* are indicated by red black boxes. Alignment results are were obtained from the website (https://www.ebi.ac.uk/Tools/msa/clustalw2/).

1.3 Supplementary Figure S3

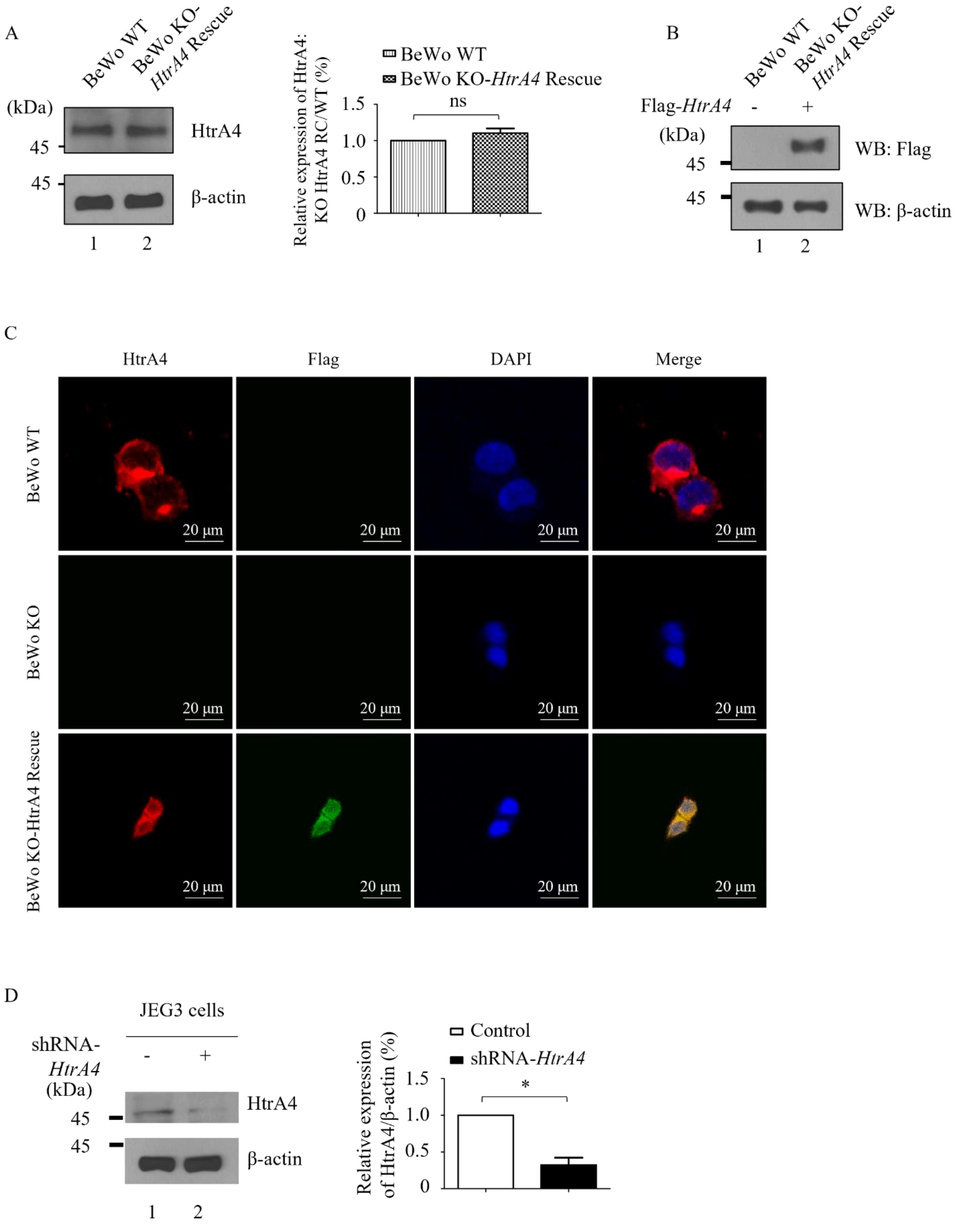

Supplementary Figure S3. Expression of HtrA4 in BeWo and JEG3 cells. BeWo KO cells were transfected with 3 μg of pcDNA3.1-*HtrA4*-Flag and incubated for 48 hrs. Cell lysates were subjected to western blot analysis to assess the expression of HtrA4 using anti-HtrA4 (A) and anti-Flag (B) antibodies. (C) The pcDNA-*HtrA4*-Flag was transfected into BeWo KO cells and incubatd for 48 hrs. The endogenous and exgenous HtrA4 were detected using anti-HtrA4 and anti-Flag antibodies to investigate the locolization of HtrA4. Nuclei were stained blue with DAPI, while endogenous HtrA4 and exogenous HtrA4 were labeled with red and green, respectively. (D) JEG3 cells were transfected with 3 μg of shRNA-*HtrA4* and incubated for 48 hrs. The cell lysates were used for Western blot analysis to confirm the expression of HtrA4. Cell lysates were used for western blot analysis to confirm the knockdown of HtrA4 expression.

Supplementary Figure S4

A

BeWo WT

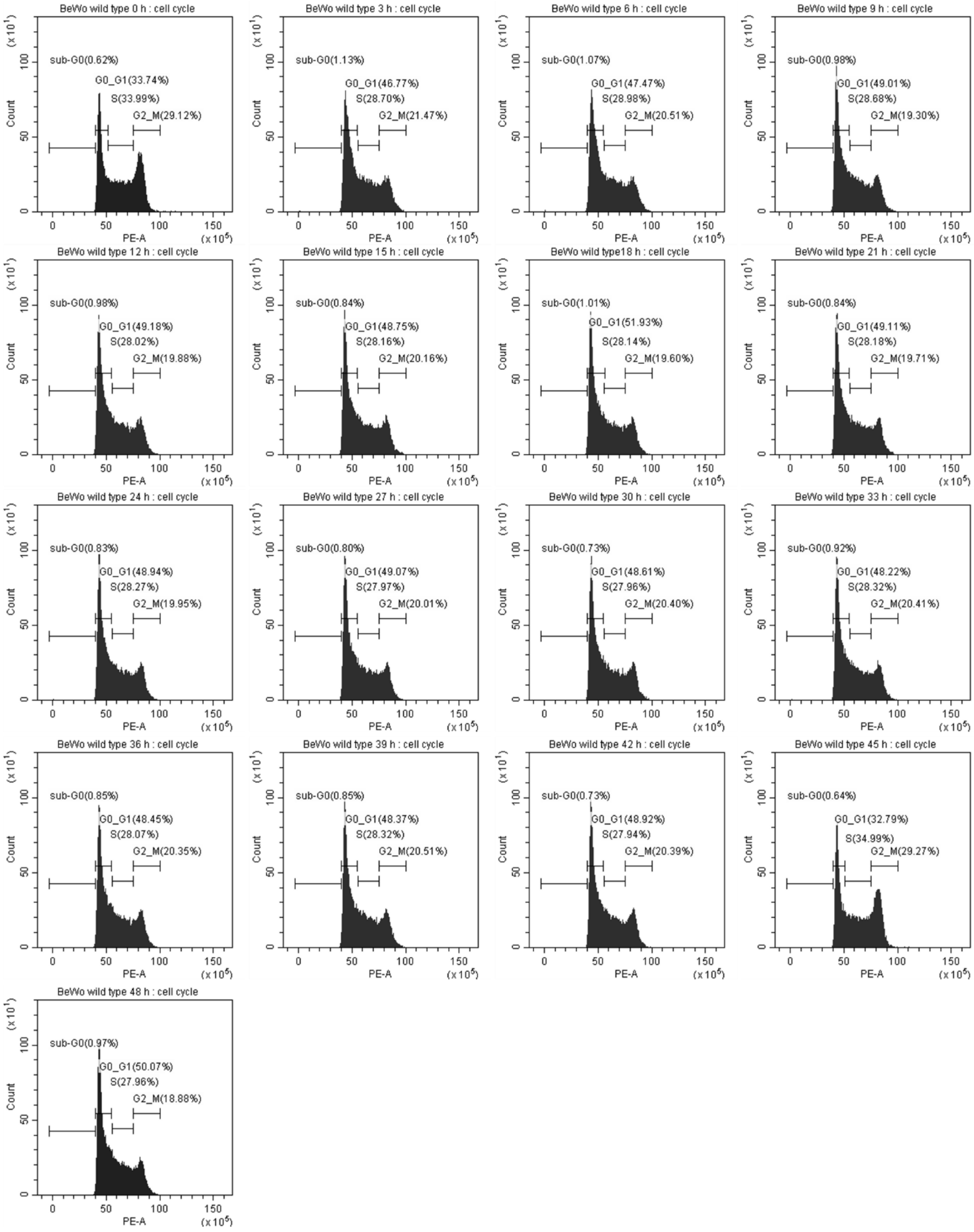

B

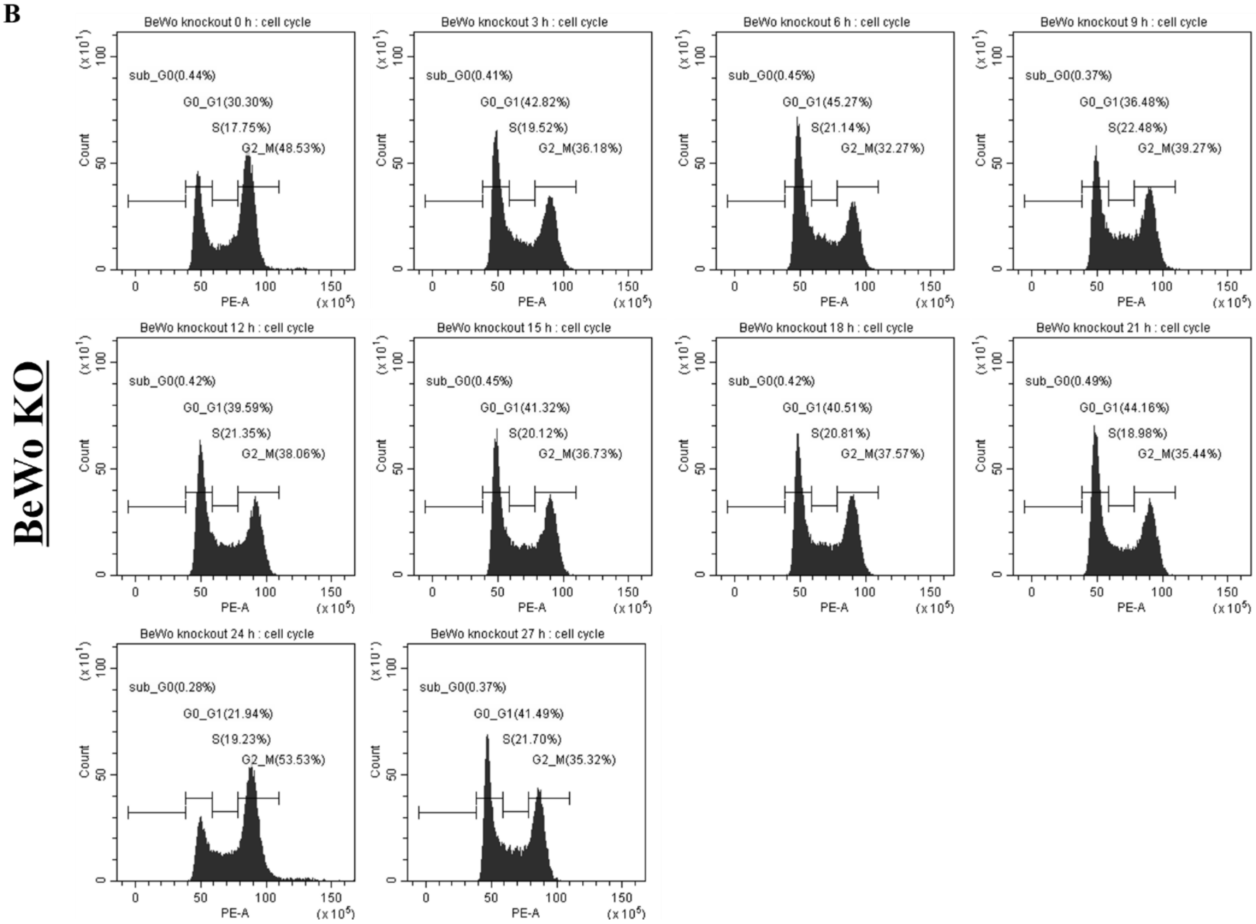

Supplementary Figure S4. Cell cycle distribution of BeWo cells at 3 hrs. (A) Representative cell cycle distribution of BeWo WT cells at 3 hrs. (B) Representative cell cycle distribution of BeWo KO cells at 3 hrs.

1.4 Supplementary Table S1

Information of designed sgRNA primers.

RGEN target (5′ to 3′)	Position	Cleavage position (%)	Direction	GC contents (%, w/o PAM)	Out-of-frame Score	Mismatches		
						0	1	2
CAG ACT TCA CGC TCG GCC GCG GG	217	22.2%	-	70.0	65.4	1	0	0

1.5 Supplementary Table S2

Information of primers for PCR and RT-PCR.

Gene	Forward sequence (5′-3′)	Reverse sequence (5′-3′)	Length (bp)
HtrA4 (T7E1)	GAG GGT TTG CAG GTC CAG	GCA CCT ACC CCA GCA TGT	436
HtrA4 (RT-PCR)	GGG GCT GAA AAG CTA CAT AC	CAC ATG CTG GGG TAG GTG	293

1.6 Supplementary Table S3

Antibodies information.

Antibody	Company	Cat. Number	Ab concentration
HtrA4	Proteintech Group	14095-1-AP	1:500
ERK1/2	Cell Signaling Technology Inc.	9194	1:1000
p-ERK1/2	Cell Signaling Technology Inc.	8544	1:1000
p38	Cell Signaling Technology Inc.	2387	1:1000
p-p38	Cell Signaling Technology Inc.	9216	1:1000
Ras	Cell Signaling Technology Inc.	3965	1:1000
Raf	Cell Signaling Technology Inc.	9422	1:1000
MKK3	CUSABIO	CSB-PA013412LA01HU	1:200
MKK6	CUSABIO	CSB-PA013415XA01DOA	1:200
Cyclin A	BD Biosciences	554174	1:200
Cyclin D1	BD Biosciences	554181	1:200

Cyclin E	BD Biosciences	554182	1:200
p-FAK(Try397)	BD Biosciences	611722	1:200
FAK	Santa Cruz Biotechnology	Sc-271195	1:200
MMP-2	Santa Cruz Biotechnology	sc-53630	1:200
MMP-9	Santa Cruz Biotechnology	sc-21733	1:200
JNK	Santa Cruz Biotechnology	sc-4061	1:200
p-JNK	Santa Cruz Biotechnology	sc-6254	1:200
β -actin	Santa Cruz Biotechnology	sc-47778	1:2000
Flag	Sigma-Aldrich	F3165	1:3000
Chorionic Gonadotropin	Invitrogen	14-6508-82	1:100
E-cadherin	Cell Signaling Technology Inc.	Sc-7870	1:100
